# Chromatin Dynamics and Gene Expression Response to Heat Exposure in Field-Conditioned versus Laboratory-Cultured *Nematostella vectensis*

**DOI:** 10.3390/ijms22147454

**Published:** 2021-07-12

**Authors:** Eviatar Weizman, Mieka Rinsky, Noa Simon-Blecher, Sarit Lampert-Karako, Orly Yaron, Ann M. Tarrant, Oren Levy

**Affiliations:** 1 Mina and Everard Goodman Faculty of Life Sciences, Bar-Ilan University, Ramat Gan 52900, Israel; miekari9@gmail.com (M.R.); blechn@gmail.com (N.S.-B.); sarit.lampert@gmail.com (S.L.-K.); orlyyaron2@gmail.com (O.Y.); 2 Department of Biology, Woods Hole Oceanographic Institution, Woods Hole, MA 02543, USA; atarrant@whoi.edu

**Keywords:** ATAC-seq, cnidarian, RNA-seq, stress response, pre-conditioning, thermal

## Abstract

Organisms’ survival is associated with the ability to respond to natural or anthropogenic environmental stressors. Frequently, these responses involve changes in gene regulation and expression, consequently altering physiology, development, or behavior. Here, we present modifications in response to heat exposure that mimics extreme summertime field conditions of lab-cultured and field-conditioned *Nematostella vectensis*. Using ATAC-seq and RNA-seq data, we found that field-conditioned animals had a more concentrated reaction to short-term thermal stress, expressed as enrichment of the DNA repair mechanism pathway. By contrast, lab animals had a more diffuse reaction that involved a larger number of differentially expressed genes and enriched pathways, including amino acid metabolism. Our results demonstrate that pre-conditioning affects the ability to respond efficiently to heat exposure in terms of both chromatin accessibility and gene expression and reinforces the importance of experimentally addressing ecological questions in the field.

## 1. Introduction

Organismal survival is dependent on an array of molecular, physiological, and behavioral responses that enable organisms to avoid or counteract harmful environmental conditions. Often, these responses to environmental stressors are complex, involving many mechanisms and interactions at cellular and organismal levels. Frequently, an organism’s response to environmental stressors, whether natural or anthropogenic, will involve changes in regulation and gene expression, which consequently alter physiology, development, or behavior. Moreover, animal origin and past growth conditions (e.g., in the field or laboratory) may change its response altogether [1].

*Nematostella vectensis*, the starlet sea anemone, is a year-round resident of estuarine environments, including salt marshes, saline lagoons, and other brackish habitats [2]. In recent years, *N. vectensis* has been established as a significant model organism for developmental [3] and genomic studies [4,5,6]. It has proven particularly informative for reconstructing the functional evolution of a subset of developmental regulatory genes for which their origin can be traced to the cnidarian-triploblast common ancestor [7,8].

**Figure 1:**
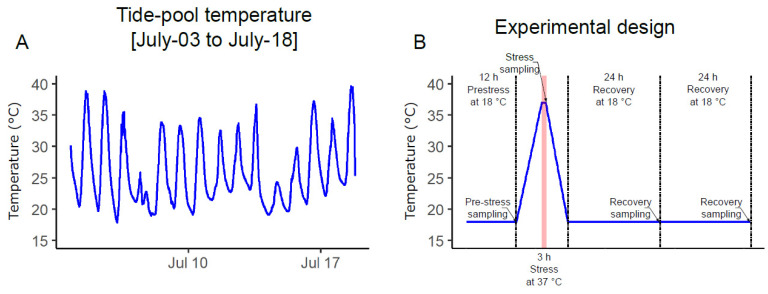
Thermal-stress experimental design. (**A**) Field temperature measurements within a tide-pool in Great Sippewissett Marsh (Massachusetts, 41°35′ N 70°39′ W) over 15 days (3 July to 18 July) representing the natural fluctuations in the field at the time of the experiment. (**B**) Schematic representation of the experiment. Field-conditioned *N. vectensis* and lab-cultured *N. vectensis* triplicates were sampled over 72 h at 4 time points, indicated by arrows; the two groups were introduced to stress of 37 °C for 3 h (marked in red) and then sampled after recovery.

Similar to many estuarine organisms, *N. vectensis* can tolerate a broad range of temperatures and other environmental variables (e.g., pH, salinity, and oxygen concentration). As a sedentary species, *N. vectensis* individuals must have broad physiological plasticity and capability to acclimate to changing environments and have been documented surviving in temperatures ranging from −1.5 °C to 41 °C [2,9] and salinities from 9 PPT to 51 PPT [2]. Moreover, large populations of *N. vectensis* can be found in tidally restricted pools that experience extreme temperature oscillations with >20 °C fluctuations daily (Figure 1A). Animals living in this varied environment are expected to develop efficient acclimation capabilities and to “learn” how to better respond to subsequent exposures to stress. For example, corals transplanted into variable thermal environments can respond better to acute heat stress than colonies that remain in more thermally stable waters [10].

Today, *N. vectensis* is accepted as a model organism with well-developed genomic tools that investigators have used to characterize mechanisms of diverse stress responses [11,12,13,14]. Nonetheless, such studies raise the question of the extent to which laboratory observations are representative of the biological responses that would be manifested by natural animal populations. It is also now clear that epigenetic mechanisms can substantially change how the genome is interpreted depending on the environment [15,16]. In other systems, understanding of these mechanisms has been facilitated by diverse approaches, including the identification of enriched transcription factor (TF) motifs within *cis*-regulatory elements (CREs) that can then reveal genes associated with a transcriptional regulatory network [17]. Hence, to gain a deeper understanding of chromatin dynamics between populations of lab-cultured and field-conditioned *N. vectensis* in response to an environmental stressor, it is important to profile the regulatory areas that are activated by the stressor in each case.

In this work, we sought to discover how the response to heat exposure is reflected in the chromatin landscape and gene expression in laboratory-cultured *N. vectensis* and field-conditioned *N. vectensis*. We hypothesized that explanting lab-cultured animals into the field (field-conditioning) would induce acclimation responses that would lead to more efficient responses to subsequent stress exposure. We found that the two populations of animals responded differently to heat exposure that mimics extreme summertime field conditions (referred to as thermal stress). While field-conditioned *N. vectensis* responded by regulating damage repair pathways, lab-cultured *N. vectensis* responded through amino acid metabolic pathways.

## 2. Results

### 2.1. Field Conditioned N. vectensis

To study the effects of acclimation to variable natural conditions on responses to subsequent heat exposure, we conducted a transplant experiment, starting with a population of *N. vectensis* that had been reared in the lab for over a year. Because *N. vectensis* can undergo asexual reproduction and have limited mobility as adults, populations are often highly clonal, particularly on small spatial scales, such as within a single tide pool [8]. A recent analysis showed that the lab population was derived of clonal and closely related individuals (e.g., 16% of pairwise comparisons represented likely clonemates; Leach, Smith, and Reitzel, personal communication). We transplanted a subset of these lab-cultured animals back into their original collection site, where they could be exposed to natural variation in temperature, light levels, salinity, food availability, and water chemistry. After one month of acclimation to field conditions, *N. vectensis* was collected at dusk (12 h prior to sampling) and transported back to the lab.

### 2.2. Chromatin Accessibility Profiles and Differential Expression Analysis of Field and Lab N. vectensis

**Figure 2:**
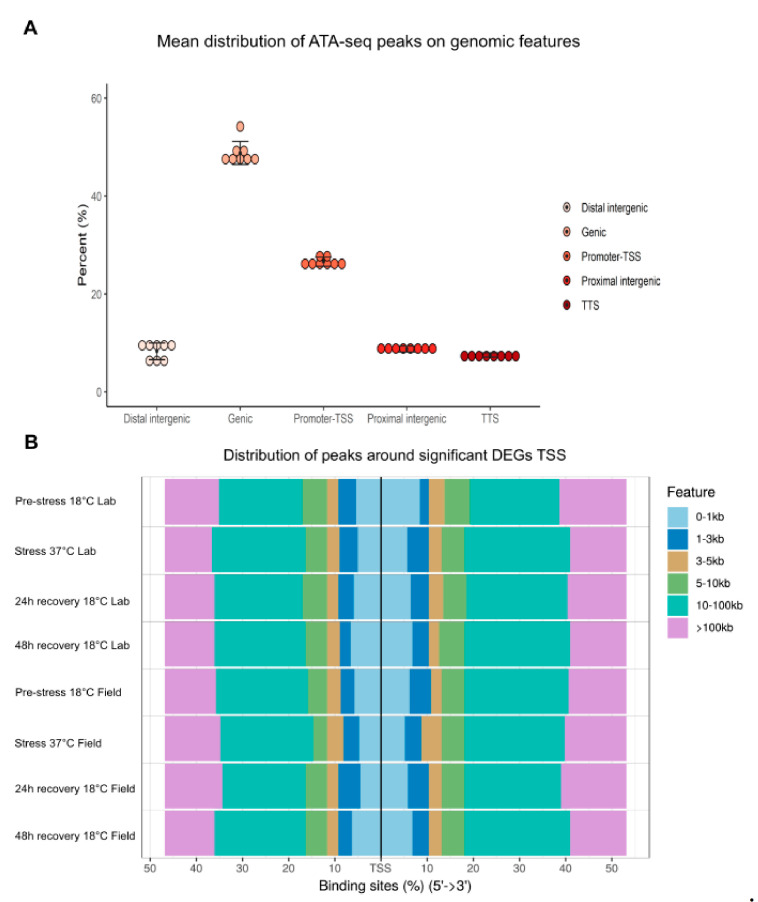
Peak distribution. (**A**) The average percentage of genomic features of field and lab *N. vectensis* calculated from all ATAC-seq libraries and sampling points (*n* = 24)—replicates were joined into one group average. Genomic features are: Genic—all ATAC-seq sites that are within the gene body (intron or exon), Promoter-TSS (Transcription Start Site)—all ATAC-seq sites that are within promoter limits (1500 bp downstream of the TSS), TTS (Transcription Termination Site)—all ATAC-seq sites that are within 500 bp upstream of the TTS, Proximal intergenic—all ATAC-seq sites that are within 1001 bp to 5000 bp downstream of the TSS or 501 bp to 5000 bp upstream of the TTS, Distal intergenic—all ATAC-seq sites that are more than 5001 upstream of the TTS or more than 5001 upstream of the TSS. (**B**) Distribution of ATAC-seq peaks around DE genes of each group.

ATAC-seq and RNA-seq experiments were conducted to compare field-conditioned *N. vectensis* and lab-cultured *N. vectensis* responses to heat exposure, simulating natural summer conditions in Great Sippewissett Marsh, Massachusetts (41°35′ N 70°39′ W) in summer 2017 (Figure 1A). We refer to the natural temperature peak (i.e., 37 °C) as the “stress” time-point. Animals were sampled at four time-points (pre-stress—18 °C, stress—37 °C, 24 h recovery—18 °C, and 48 h recovery—18 °C, Figure 1B) from both field-conditioned and lab-cultured groups. After sampling, ATAC-seq was applied to sensitively measure high-resolution chromatin accessibility. Libraries were sequenced from three independent biological replicates from each group and sampling point. We obtained an average alignment to the *N. vectensis* genome of 86.2% and an average alignment to non-nucleic DNA of 3.3% (Table 1). The biological replicates were highly similar, demonstrating highly reproducible data from *N. vectensis* whole animal sampling (Table 2). We identified a median of 84,080 significant peaks per sample (max: 133,764, min: 41,212), representing accessible DNA sites (Table S1). In addition, distribution profiles of ATAC-seq peaks over the *Nematostella* genome revealed that ~9% of peaks are within 3000 bp upstream to transcriptional start sites (TSS) (Figure 2A).

Next, in order to discover changes in gene expression in the field and lab *N. vectensis* groups, we performed differential expression analysis of our RNA-seq data. Transcriptome sequencing produced >475 million reads with an average of 25 million reads per sample (SD ± 2). After filtering, we obtained >80% alignment efficiency to the *N. vectensis* published genome (Genome version: Nemvec1), with reads mapped to a total of 25,564 genes. The mapped reads from each sample were converted to counts per gene and were processed by a generalized model in DESeq2 to identify unique differentially expressed (DE) genes within each thermal treatment. We observed that at the stress time point, a total of 3332 genes were DE relative to the pre-stress time-point (Table S2). Further, we found that the ATAC-seq peak distribution around significantly DE genes is enriched around promoters, indicating higher accessibility in these regions (Figure 2B).

### 2.3. Rearing Conditions Affect TF Enrichment and Transcriptional Response during Heat Exposure

**Figure 3:**
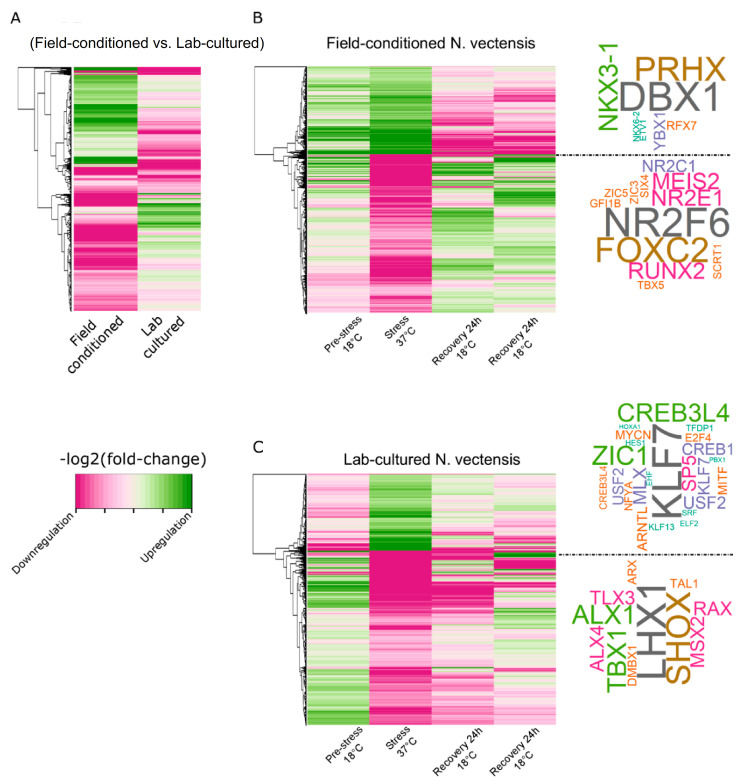
Rearing conditions affect TF enrichment and transcriptional response during heat exposure. (**A**) DE gene profile (*n* = 3332 genes obtained from both groups) at the stress time point relative to the corresponding pre-stress time point (3-h at 37°C). (**B**,**C**, Left) Heatmaps of [–log_2_(fold-change)] of RNA expression of *N. vectensis* genes from field-conditioned (**B**, *n* = 2572) and lab-cultured (**C**, *n* = 887) groups. (**B**,**C**, Right) Word clouds clustering names of transcription factors most significantly enriched in accessible chromatin (ATAC-seq) within promoters of differentially expressed genes (RNA-seq stress vs. pre-stress control).

The RNA-seq experiment revealed different expression profiles of each *N. vectensis* group (field-conditioned vs. lab-cultured). We identified 2572 DE genes in the field stress group relative to the pre-stress field control (Pre-stress 18 °C Field), and 887 DE genes in the lab stress group relative to the pre-stress lab control (Pre-stress 18 °C Lab), with only 127 overlapping genes (Figure 3 and Figure S1). Overall, the most differentially expressed genes, including many of the 127 overlapping genes, showed an opposite direction of regulation between the two groups (Figure 3A). Interestingly, genes upregulated by heat exposure in the field-conditioned *N. vectensis* group were upregulated during pre-stress (relative to the lab-cultured group) and maintained that upregulation during stress. In contrast, upregulated genes of the lab-cultured *N. vectensis* group during pre-stress clustered in an opposite pattern relative to the stress period. Moreover, the gene expression pattern in the field-conditioned group was maintained throughout the recovery period, while the lab *N. vectensis* group seemed to have an irregular expression pattern over time in response to the heat exposure (Figure 3B,C). To learn more about the regulatory factors that control and direct these differences, we performed motif enrichment analysis on promoters of DE genes (defined as 1500 bp upstream of TSS). Within the accessible upstream sequence (from ATAC-seq, discussed further below) of genes upregulated during thermal stress in field-conditioned *N. vectensis*, we found enrichment of *DBX1*, *PRHX*, *NKX3-1*, *YBX1*, *RFX7*, *NKX6-2*, and *ETV1* motifs, and promoters of downregulated genes during stress were enriched with *NR2F6*, *FOXC2*, *RUNX2*, *MEIS2*, *NR2E1*, *NR2C1*, *GFI1B*, *TBX5*, *SCRT1*, *ZIC3*, *ZIC5*, and *SIX4* motifs (Figure 3B). In the promoters of genes upregulated during thermal stress in lab-cultured *N. vectensis*, we found enrichment of *KLF7*, *ZIC1*, *CREB3L4*, *SP5*, *MLX*, *USF2*, *CREB1*, *ARNTL*, *MYCN*, *MITF*, *E2F4*, *NFYA*, *KLF13*, *TFDP1*, *EHF*, *HES1*, *ELF2*, *SRF*, *HOXA1*, and *PBX1* motifs, and promoters of downregulated genes during stress were enriched with *LHX1*, *SHOX*, *TBX1*, *ALX1*, *RAX*, *ALX4*, *TLX3*, *MSX2*, *ARX*, *DMBX1*, and *TAL1* motifs (Figure 3C). Many of the TFs associated with the enriched motifs are active in different stress response pathways; 26.32% of TF motifs found in field-conditioned and 22.58% of TF motifs found in lab-cultured *N. vectensis* are directly stress-related; the largely non-overlapping composition of these lists emphasizes the different response of each group to heat elevation (Figure S2 and Table S1).

### 2.4. Changes in DNA Regulatory Landscape in Response to Heat Exposure

**Figure 4:**
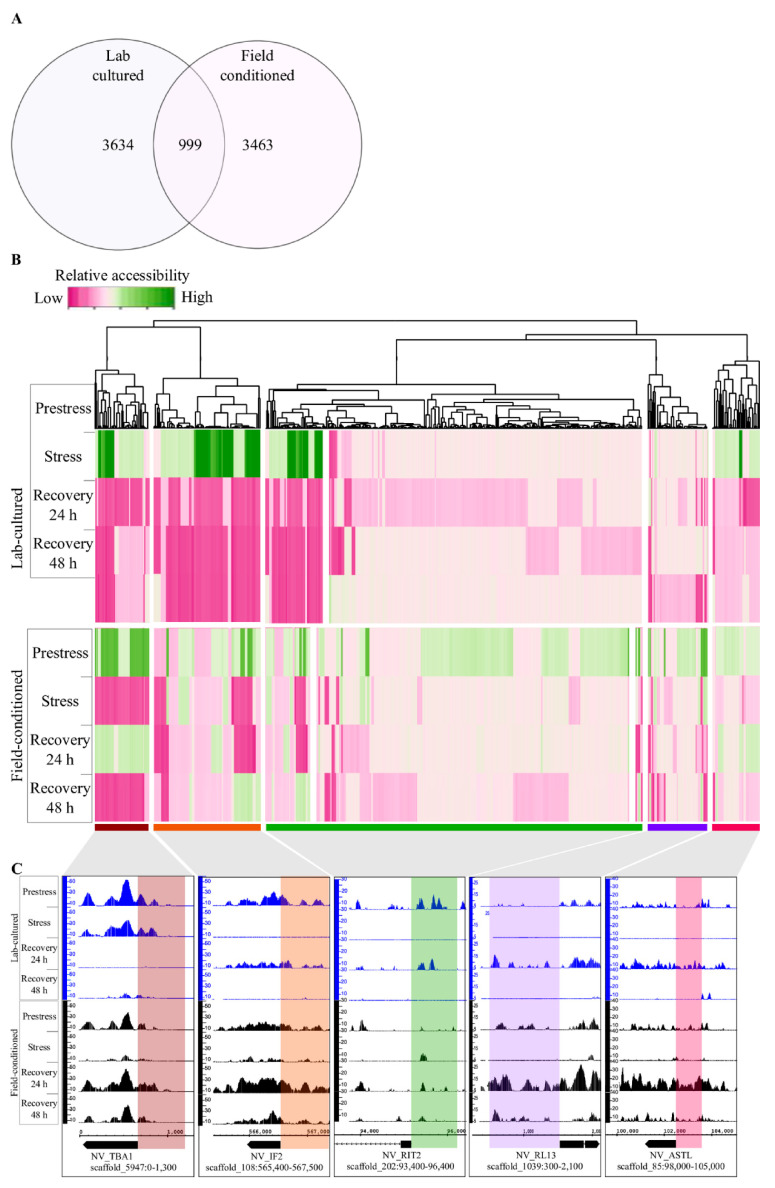
Changes in DNA regulatory landscape in response to heat exposure. (**A**) Venn diagram of 4462 differentially accessible promoters in the field-conditioned group (purple), and 4633 differentially accessible promoters in the lab-cultured *N. vectensis*. (**B**) Heatmap of 999 ATAC-seq promoter sites found in both groups as identified for each thermal treatment were clustered, yielding five accessibility clusters: cluster (1)—brown, cluster (2)—orange, cluster (3)—green, cluster (4)—purple, and cluster (5)—pink. Each column represents one gene in lab-cultured (top) and field-conditioned (bottom) groups. (**C**) Representative examples of genes from each accessibility cluster—blue peaks represent lab-cultured ATAC-seq peaks, and black peaks represent field-conditioned ATAC-seq peaks. Specific coordinates are shown below tracks; sites of interest are highlighted in a similar color representing the 1000 bp promoter region of each gene.

The regulatory DNA landscape in response to heat exposure was mapped to display promoter sites that changed accessibility in response to the thermal stress in both field-conditioned and lab-cultured *N. vectensis.* We identified 4462 heat-responsive sites in field-conditioned *N. vectensis* and 4633 heat-responsive sites in lab-cultured *N. vectensis*, with 999 overlapping sites (Figure 4A and Figure S3 and Table S3). Even sites that were regulated by heat in both groups exhibited different accessibility patterns over time from pre-stress through recovery (Figure 4B,C). This observation suggests that the regulatory response pathways depend on the pre-conditioning of the animals to lab or field environments.

### 2.5. Enrichment Analysis Revealed Different Response Pathways

**Figure 5:**
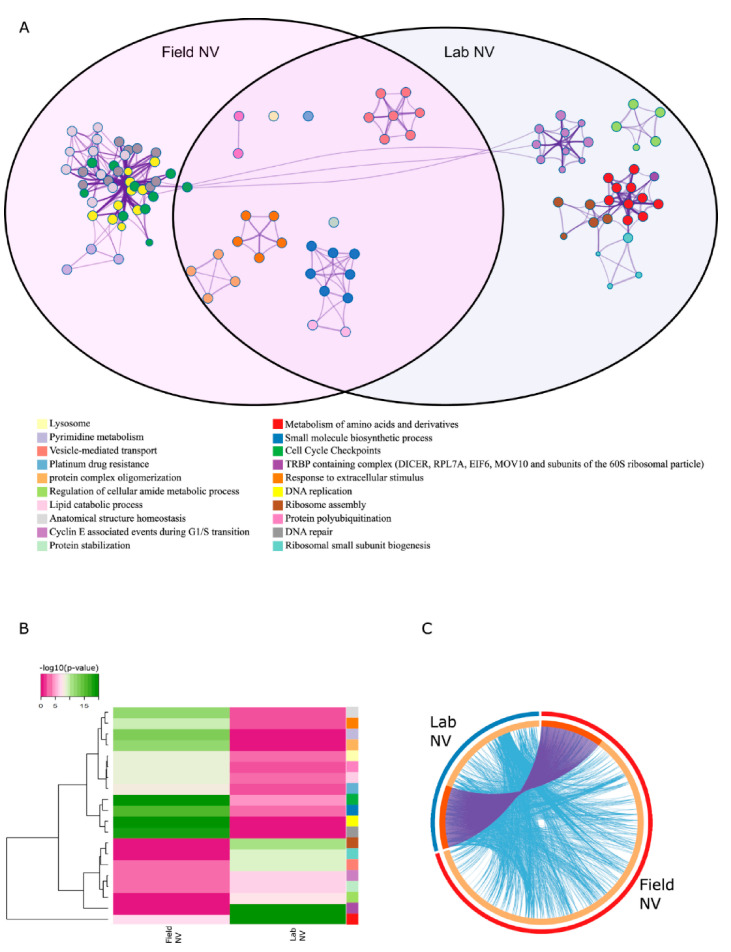
Enrichment analysis revealed different response pathways. (**A**) A subset of enriched KEGG pathways, gene ontology biological processes, gene ontology molecular functions, reactome gene sets, canonical pathways, and CORUM biological processes displayed in a network. Terms with a similarity score > 0.3 are linked by an edge (the thickness of the edge represents the similarity score). Each term is represented by a circular node, where the size is proportional to the number of input genes included in that term, and the color represents the cluster’s identity. Term labels are only shown for one representative term per cluster. Purple shading contains terms overrepresented in DE genes in the stress vs. pre-stress comparison of field-conditioned *N. vectensis*. Blue shading contains terms overrepresented in DE genes in the stress vs. pre-stress comparison of lab-cultured *N. vectensis*. (**B**) Heatmap representing enriched KEGG pathways, gene ontology biological processes, gene ontology molecular function, reactome gene sets, canonical pathways, and CORUM biological processes of the *N. vectensis* heat exposure response. The colors in the heat map represent statistical significance (−log10(P) value). The colors along the sides correspond to the legend above. (**C**) Circos plot describing overlaps in the heat exposure response (stress vs. pre-stress) between the two *N. vectensis* groups. The outermost circles are proportional to the number of enriched measured transcripts within each *N. vectensis* group. Inner circles indicate the proportion of measured transcripts unique to each group (light orange) and shared between groups (dark orange). Interconnecting purple lines indicate shared interacting measured transcripts, and interconnecting light blue lines indicate shared gene ontology terms between interacting measured transcripts.

Enrichment analysis of DE genes in both field-conditioned *N. vectensis* and lab-cultured *N. vectensis* across all experimental sampling points revealed different KEGG pathways, gene ontology biological processes (BP), gene ontology molecular functions (MF), reactome gene sets, canonical pathways, and CORUM biological processes that are enriched within each group in response to heat exposure (Figure 5 and Tables S4 and S5). DE genes in the field-conditioned *N. vectensis* were enriched with pathways and processes related to cell cycle checkpoints, DNA replication and repair, and structure homeostasis. DE genes in lab-cultured *N. vectensis* were enriched with pathways and processes related to metabolic processes, such as metabolism of amino acids, regulation of cellular amide metabolic process, vesical mediated transport, and protein stabilization. Taken together, functional analyses of the composition of differentially expressed genes and comparison of associated regulatory factors identified distinct responses of the two *N. vectensis* groups to heat exposure.

## 3. Discussion

The ecological relevance of lab-based studies is frequently debated. On the one hand, investigations with laboratory-cultured animals have provided wide-ranging and detailed insights into biochemical, physiological, and behavioral processes [1]. On the other hand, removing animals from the context of their natural environment might alter results and observations dramatically. For example, wild and captive European starlings (*Sturnus vulgaris*) exhibit different hormonal responses to chronic stress [18]. In addition, studies in diverse animals have revealed differences in circadian behavioral patterns between wild and captive populations [19,20,21]. These examples and many other similar results emphasize the importance of re-introducing biological questions to the “real world.”

The heat stress response is a widely studied topic due to concerns about global warming and its effects on many marine animals and specifically cnidarians. Great effort has been invested in researching this phenomenon to predict probable outcomes. In its natural habitat, *N. vectensis* is exposed to fluctuations in a wide range of factors that can lead to stress, such as salinity and temperature. Adaptation to these types of environments has presumably selected for physiological tolerance and plasticity in *N. vectensis,* and thus, this species serves as an interesting model of cnidarian responses to environmental stressors [12,13]. Nevertheless, we questioned the validity of strictly lab-based experiments to characterize the responses of these animals to natural stressors.

**Figure 6:**
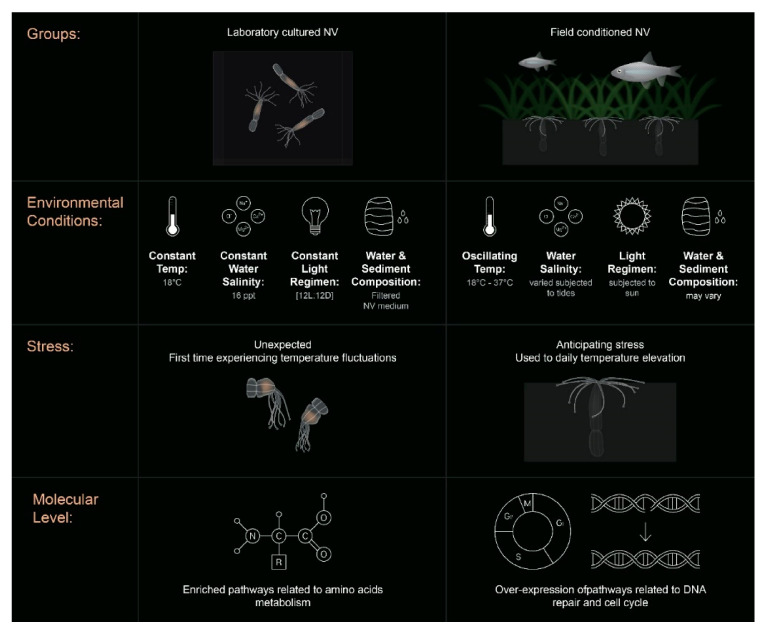
Conceptual summary. A scheme summarizing the different conditions experienced by lab-cultured *N. vectensis* (**left**) and field-conditioned *N. vectensis* (**right**) and our results. Among the conditions shown, the lab-cultured anemones were reared in diluted, filtered seawater (NV medium), and field-conditioned animals were exposed to sediments and naturally variable water chemistry.

In this work, we applied RNA-seq and ATAC-seq to investigate the responses of laboratory-cultured versus field-conditioned *N. vectensis* to experimental conditions mimicking the daily temperature rise experienced within a temperate tide-pool during summer 2017 (Figure 1) and to observe the different expression and regulatory profiles of the two groups. A conceptual summary of the different conditions experienced by the two groups and our results is presented in Figure 6. We observed different transcriptional responses in each group; moreover, when we examined the stress time-point in comparison to pre-stress controls, most of the DE genes were regulated in opposite directions between the two groups (Figure 3A). This suggests that field conditions alter animal responses in comparison to rearing in a strictly controlled lab environment. These expression profiles are a product of dissimilar regulatory networks of TFs activated within each group, as revealed from analyzing the chromatin accessible landscape (Figure 2 and Figure 3). Yet, there is little knowledge about the relationship between promoter accessibility and its related gene expression, as various regulatory mechanisms can regulate cnidarian gene activity. For example, we have previously shown that clock-controlled genes present different accessibility patterns over a 24 h period [22]. It was found that promoters of some clock-controlled genes were constantly accessible, whereas the accessibility of others oscillated [22]. Further research is needed in the field of epigenetics and, more specifically, on chromatin spatial interactions, in order to elucidate how promoter accessibility is linked with changes in gene expression in cnidarians.

In the marsh habitat, daily temperature oscillations are periodic and, therefore, can be anticipated by the resident animals [23,24]. On the other hand, lab-cultured animals are subjected to a strictly controlled environment with a regular light cycle and constant temperature and salinity; thus, they may never experience stress throughout their whole life cycle and even generations back. Although many features differed between the laboratory and field environments, we postulate that the major differences presented between the groups are due to the previous acclimation of field-conditioned *N. vectensis* to daily temperature fluctuations. Accordingly, the GO enrichment analysis showed that, in response to heat exposure, field-conditioned *N. vectensis* had an enriched network of pathways related to DNA repair and homeostasis, such as cell cycle checkpoints, DNA replication and repair, and structure homeostasis (Figure 4A). In contrast, laboratory *N. vectensis* activated a more diffuse network that was enriched with metabolic processes such as metabolism of amino acids, regulation of cellular amide metabolic process, vesical mediated transport, and protein stabilization; these pathways are related to the protein breakdown that can be induced during heat stress [25,26]. Similarly, two distinct responses were classified in the genus *Acropora* using meta-analysis of gene expression responses in 14 published studies [27]. The researchers identified the first “type A” response as an environmental stress response (ESR) to severely stressful conditions, while the “type B” response was more variable and was identified under less severe stress conditions [27]. Therefore, we infer that pre-conditioning *N. vectensis* to natural field versus lab environments leads to different types of responses to heat exposure. The fact that fewer functional pathways responded to the heat exposure in the field group leads us to postulate an anticipation mechanism in which the response to the exogenous cues is more accurate and efficient. We would predict to observe a similar anticipation mechanism within wild *N. vectensis* collected directly from the field. However, additional field-based studies are needed to determine the extent to which thermal stress responses of natural populations of *N. vectensis* are predictable.

In conclusion, in this work, we characterized the discordant response of laboratory-cultured versus field-conditioned animals to identical heat exposure (Figure 5). Our approach allowed us to examine the effects of acclimation to a variable natural environment on the response to a single controlled stressor. In this context, field-conditioned *N. vectensis* were accustomed to highly variable temperatures and had a more focused molecular response, as indicated by a tighter network of enriched GO terms. To advance our mechanistic understanding of different responses, we often aspire to perform strictly controlled lab experiments. Although such lab experiments are very informative, understanding and predicting animals’ responses to stress events can be improved by including more realistic pre-exposure conditions. This study highlighted the value of conducting experiments in an environmental context, focusing particularly on the role of acclimation to naturally variable environments. Effects of environmental acclimation on temperature responses can be further explored through structured experimental approaches including reciprocal transplant experiments and comparison of stress responses across natural environmental clines. Integration of these strictly controlled and environmentally realistic approaches is necessary for building a rich and robust understanding of stress responses during the Anthropocene.

## 4. Limitations of the Study

This study discusses the importance of experimentally addressing ecological questions in the field; thus, sampling wild anemones retrieved from the marshes to compare with lab-cultured anemones would confirm that the differences presented between the groups are due to the previous acclimation of field-conditioned *N. vectensis* to daily temperature fluctuations. Accordingly, we could not determine whether the one-month conditioning period was sufficient to completely re-wire gene regulatory networks to match what will be expected in organisms reared in the field. In addition, we propose that thermal variability in the field environment was a major driver of the differences in the thermal stress responses between the two groups. This hypothesis could be directly tested by simulating thermal variability in the lab.

## 5. Materials and Methods

### 5.1. Animal Culture

All *N. vectensis* used in the experiments were derived from a laboratory culture originally established with animals from Great Sippewissett Marsh (41°35′ N 70°39′ W) in 2002. The laboratory population was periodically supplemented with additional field collections. The portion of the laboratory population used for experiments had been in culture for at least one year. For the experiments, adult *N. vectensis* (1–3 cm, when fully extended) were kept in glass bowls filled with 200 mL of filtered natural seawater at a salinity of 15 PSU, under full-spectrum light provided by “Reef Sun” bulbs (Zoo Med Laboratories, San Luis Obispo, CA, USA) and a constant temperature of 18 °C. Twenty individuals were kept in each container in a static water system. The animals were fed five times a week with freshly hatched brine shrimp (*Artemia* sp. nauplii). To create a field-conditioned *N. vectensis* group with sufficient animals for sampling, we deployed the animals into a tide pool known to house a native population (within Great Sippewissett Marsh, Massachusetts) in mesh-covered containers [28]. These animals were acclimated to field conditions in the marsh for one month. Of the 100 *N. vectensis* that were deployed in the field, 60 were recovered after one month. While we do not know if the other 40 animals did not survive or escaped from the cages, a few were collected with half of their body on each side of the mesh, so we suspect that escape was possible.

### 5.2. Experimental Design

*N. vectensis* was collected from the field at dusk (when the measured water temperature in the tide-pool reached 18 °C) 12 h prior to the pre-stress sampling and transported back to the lab in containers that included marsh water and sediment to minimize collection stress. The animals were isolated from the sediment into filtered seawater and were immediately incubated in lab conditions (light and temperature). The animals were sampled for ATAC-seq and RNA-seq experiments at 4 time points (Figure 1B). Biological triplicates (three replicates containing one whole animal each) were sampled and processed as described in the “ATAC-seq nuclear isolation and library preparation” section below. Stress was induced by incubating the containers in a water bath that was monitored with a digital thermometer. Temperature was manually adjusted over 6 h, increasing by ~1.5 °C every half an hour from 18 °C to 37 °C, then the animals were held at 37 °C for three more hours. Immediately after sampling the stress time point, we gradually decreased the temperature over 6 h, lowering it by ~1.5 °C every half an hour from 37 °C to 18 °C. The animals were then kept in constant conditions for 48 h.

### 5.3. ATAC-Seq Nuclear Isolation and Library Preparation

Nuclei were isolated from adult *N. vectensis* during each sampling point. From each sample, a tissue was suspended in 500 µL PBS-NAC 2% (N-acetyl-cysteine, Sigma-Aldrich, St. Louis, MO, USA) by pipetting in a 1.5 mL microcentrifuge tube [29]. The suspension was centrifuged at 1500× *g* for 5 min at 4 °C. The pellet was re-suspended in 500 µL PBS, and the cells were counted. The 400,000 cells were then re-suspended in 500 µL PBS and centrifuged at 1500× *g* for 5 min at 4 °C. The pellet was suspended in 50 µL of ATAC-seq lysis buffer (10 mM TRIS-Cl pH 7.4, 10 mM NaCl, 3 mM MgCl_2_, 0.1% IGEPAL CA630) and centrifuged at 300× *g* for 10 min at 4 °C. The supernatant was collected and kept in a 1.5 mL tube on ice. The pellet was re-suspended in 50 µL and centrifuged at 300× *g* for 10 min at 4 °C. The supernatant was combined with the supernatant from the previous step. Then, 9 µL of isolated nuclei were stained with DAPI to verify the isolation of intact nuclei. The isolated nuclei were then centrifuged at 1500× *g* for 10 min at 4 °C. Immediately following this centrifuge step, the pellet was re-suspended in the transposase reaction mix (25 µL 2× TD buffer, 2.5 µL transposase (Illumina REF: 15028212) and 22.5 µL nuclease-free water). The transposition reaction was carried out for 30 min at 37 °C. Directly following transposition, the sample was purified using an Invitrogen Pure-Link PCR purification kit (Thermo Fisher Scientific, Waltham, MA, USA; REF: K310001). Following purification, library fragments were amplified using 1× NEB-Next PCR master mix (New England Biolabs, Ipswitch, MA, USA; M0541S) and 1.25 µM of custom Nextera PCR primers forward and reverse, using the following PCR conditions: 72 °C for 5 min; 98 °C for 30 s; and a variable number of cycles as needed (we added 4–12 cycles) at 98 °C for 10 s, 63 °C for 30 s, and 72 °C for 1 min. To reduce GC and size bias in our PCR, we monitored the PCR reactions using qPCR to stop amplification before saturation. To do this, we amplified the full libraries for five cycles, after which we took a 4 µL aliquot of the PCR reaction and added 6 µL of the PCR cocktail with Sybr Green (Promega, Madison WI, USA; REF: A6001) at a final concentration of 0.6×. We ran this reaction for 20 cycles to determine the additional number of cycles needed for the remaining 46 µL reaction. The libraries were purified using Agencourt AMPure XP beads (Beckman Coulter, Indianapolis, IN, USA; Cat. No. 63881) and analyzed on a Tape-Station. The primers used to amplify ATAC-seq libraries are listed in Table S6.

### 5.4. RNA-Seq Library Preparation

Total RNA was extracted from samples (three replicates per sampling point, each containing one whole animal) using the TRIzol reagent (Thermo Fisher Scientific, Waltham, MA, USA) and a modified version of the manufacturer’s protocol that included an additional chloroform extraction and magnesium chloride precipitation overnight. The animals were lysed in 0.3 mL TRIzol and transferred to a 2 mL tube containing an extra 0.7 mL TRIzol. The tubes were then incubated for 30 min at room temperature followed by a 20 min centrifuge at × *g* at 4 °C to eliminate residual tissue fragments. 1000 µL of the remaining upper supernatant was transferred to a 1.5 mL tube with 300 µL of chloroform, shaken vigorously, and kept at room temperature for 10 min, followed by centrifuging at 12,000× *g* for 15 min at 4 °C. After this centrifugation, there were two visible phases. A quantity of 800 µL of the aqueous phase was transferred to a 1.5 mL tube containing 600 µL of chilled isopropanol. Following 10-min incubation at room temperature, the tubes were centrifuged at 12,000× *g* for 10 min at 4 °C. The remaining supernatant was removed, and the visible pellet was washed with 1 mL of 75% ethanol and then centrifuged at 7500× *g* for 5 min at 4°C. The last step of the ethanol wash was performed a second time and was followed by removing the ethanol and air drying the tubes in a clean chemical hood. The dry pellets were resuspended in 40 µL RNAse free water and incubated at 57 °C for about 5 min until the pellet dissolved. Purified RNA samples were analyzed using a Nano-Drop 1000 spectrophotometer (Thermo Fisher Scientific, Waltham, MA, USA) to assess RNA quantity and 2100 Bioanalyzer (Agilent, Santa Clara, CA, USA) to assess RNA quality (RIN > 8.5). RNA samples with integrity values (RINs) > 8.5 were used for deep sequencing analyses. RNAseq libraries were pooled and prepared from 1.5-μg aliquots of RNA for each treatment and group, using the Illumina NEB ultra II RNA Library Preparation Kit v2 kit, according to the manufacturer’s protocol.

### 5.5. Data Analysis

ATAC-seq and RNA-seq libraries were single-end (SE) sequenced (read length was 75 bp) using Illumina NextSeq. Quality of ATAC-seq reads was assessed using fastQC and multiQC algorithms. Reads were aligned to the Nemve1 *N. vectensis* genome using bowtie2 [30]. Peaks were called by applying MACS2 [31], with the following parameters: -g 450000000 --nomodel --extsize 75 --shift -30. TF-binding motifs enrichment was identified within the promoters (1500 bp downstream to TSS) peaks using AME [32]: ame --verbose 1 --oc --scoring avg --method fisher --hit-lo-fraction 0.25 --evalue-report-threshold 10.0 db/CIS-BP/*Nematostella*\_vectensis.meme. Further analysis was performed using BEDTools suites [33] (bedtools bamtobed, bedtools shift, bedtools merge, bedtools jaccard, bedtools intersect, bedtools window, bedtools getfasta) with default parameters and custom R scripts.

Quality of RNA-Seq reads was assessed using fastQC and multiQC algorithms. Reads were aligned to the Nemve1 *N. vectensis* genome using STAR aligner [34]. Read count results (obtained using HTseq) were analyzed using the R package DEseq2 (v 1.22.2) to detect statistically distinct expression of genes, requiring a *p*-value of ≤0.05 and |−log2(fold-change)| ≥ 0.6 (at least 1.5-fold change). Heatmaps of all the significantly accessible/expressed genes were generated using the heatmap.2 function from the R BIOCONDUCTOR package GPLOTS (v2.17.0). Heatmap.2 was used with the default clustering method and scaling the data by rows. GO terms for biological processes, KEGG pathways, and gene promoters found within treatment specific peaks were defined, and were subsequently analyzed for enriched terms using metascape.org with a threshold of 1 × 10^−2^ [35].

## Figures and Tables

**Table 1:** Alignment rates of ATAC-seq reads from *N. vectensis* samples to the nuclear genome and mitochondrial DNA.

Sample Name	% Alignment to Genome	% Alignment to Mitochondrial DNA
Pre-stress 18 °C Lab (rep-1)	87.44%	0.68%
Pre-stress 18 °C Lab (rep-2)	85.15%	0.71%
Pre-stress 18 °C Lab (rep-3)	90.48%	0.13%
Pre-stress 18 °C Field (rep-1)	84.93%	5.72%
Pre-stress 18 °C Field (rep-2)	88.32%	7.96%
Pre-stress 18 °C Field (rep-3)	89.85%	11.01%
Stress 37 °C Lab (rep-1)	85.89%	2.11%
Stress 37 °C Lab (rep-2)	86.67%	1.07%
Stress 37 °C Lab (rep-3)	89.71%	1.33%
Stress 37 °C Field (rep-1)	82.25%	2.11%
Stress 37 °C Field (rep-2)	89.23%	11.34%
Stress 37 °C Field (rep-3)	86.49%	13.93%
24 h recovery 18 °C Lab (rep-1)	91.37%	0.93%
24 h recovery 18 °C Lab (rep-2)	91.85%	3.45%
24 h recovery 18 °C Lab (rep-3)	82.26%	1.26%
24 h recovery 18 °C Field (rep-1)	89.70%	4.72%
24 h recovery 18 °C Field (rep-2)	84.97%	6.66%
24 h recovery 18 °C Field (rep-3)	87.60%	3.47%
48 h recovery 18 °C Lab (rep-1)	86.23%	1.35%
48 h recovery 18 °C Lab (rep-2)	92.05%	1.03%
48 h recovery 18 °C Lab (rep-3)	89.55%	1.06%
48 h recovery 18 °C Field (rep-1)	83.37%	5.13%
48 h recovery 18 °C Field (rep-2)	89.31%	5.10%
48 h recovery 18 °C Field (rep-3)	82.38%	0.89%

**Table 2:** Pearson correlation (R^2^) results * of pairwise comparisons between replicates in each treatment.

Sample Name	Rep 1 vs. Rep 2	Rep 2 vs. Rep 3	Rep 1 vs. Rep 3
Pre-stress 18 °C Lab	0.868	0.826	0.952
Pre-stress 18 °C Field	0.983	0.989	0.999
Stress 37 °C Lab	0.845	0.847	0.865
Stress 37 °C Field	0.815	0.746	0.83
24 h recovery 18 °C Lab	0.805	0.816	0.789
24 h recovery 18 °C Field	0.852	0.821	0.879
48 h recovery 18 °C Lab	0.702	0.702	0.902
48 h recovery 18 °C Field	0.959	0.191	0.17

* All correlations are significant at the 0.01 level (2-tailed).

## Data Availability

The sequencing data reported in this study has been deposited to the Sequence Read Archive (SRA) BioProject, under accession: PRJNA598421. ATAC-seq peak data is available at: 10.6084/m9.figshare.14909490.

## Supplementary Materials

The following are available online at https://www.mdpi.com/article/10.3390/ijms22147454/s1.
