# Supplementary material for: Chromatin Dynamics and Gene Expression Response to Heat Exposure in Field-Conditioned versus Laboratory-Cultured Nematostella vectensis
Source: Int J Mol Sci. 2021 Jul 12;22(14):7454. doi: 10.3390/ijms22147454 (PMC8303994; doi:10.3390/ijms22147454)
Supplement: Supplementary file 1 [file ijms-22-07454-s001.zip › supplementary.pdf]

Suplamentary figures:

Table S1. Number of peaks detected per sample using MACS2.

Sample Name	# of peaks in rep 1	# of peaks in rep 2	# of peaks in rep 1
Pre-stress 18 °C Lab	104756	133764	112986
Pre-stress 18 °C Field	78973	68032	62041
Stress 37 °C Lab	41212	76922	83081
Stress 37 °C Field	55382	72263	115248
24h recovery 18 °C Lab	86776	84199	87249
24h recovery 18 °C Field	118525	89064	100770
48h recovery 18 °C Lab	80117	100069	121582
48h recovery 18 °C Field	78651	83962	73280

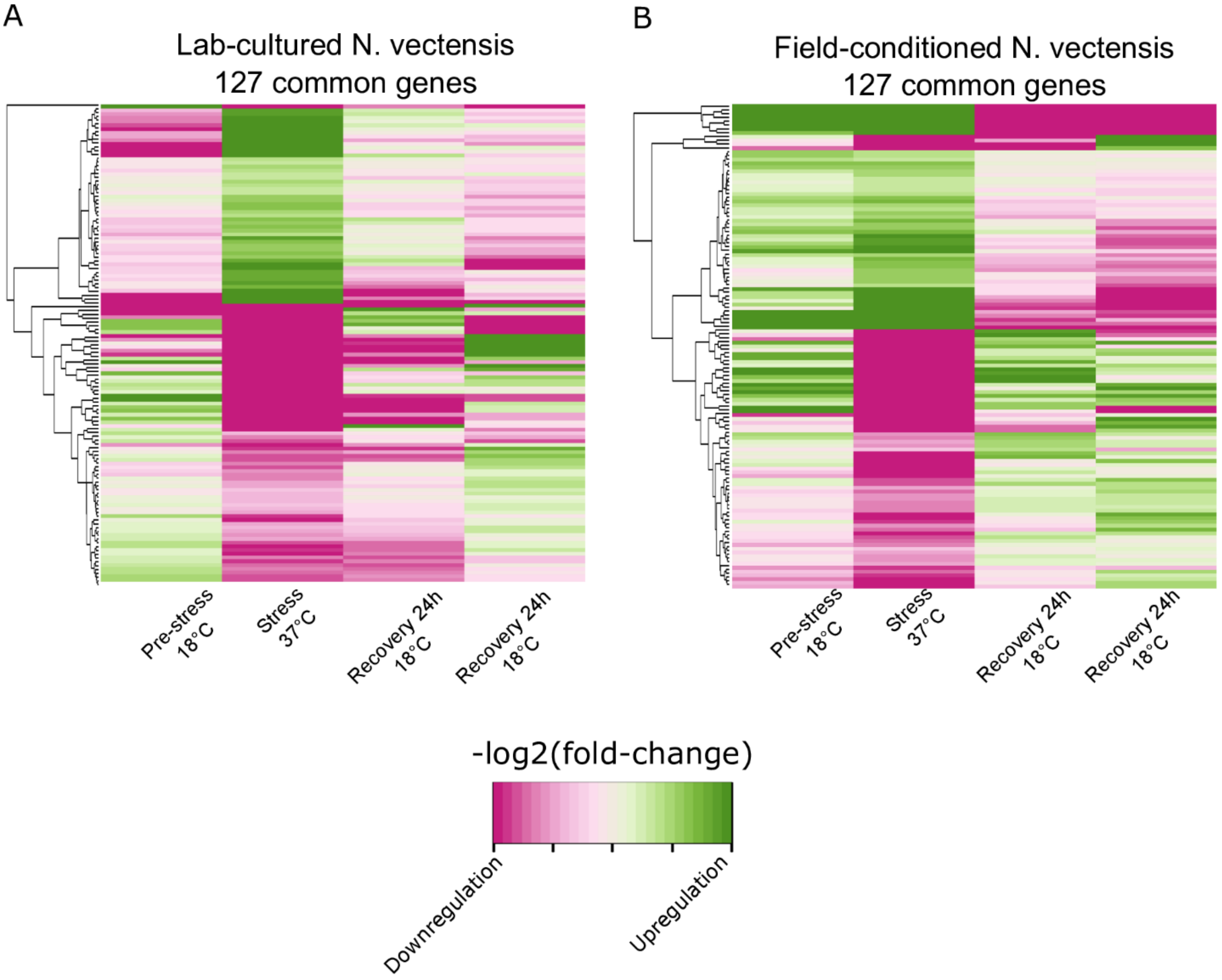

Figure S1: Rearing conditions effect transcriptional response during heat exposure. Heatmaps of –$\log_2$(fold-change) of RNA expression of *N. vectensis* 127 overlapping genes from lab-cultured and field-conditioned groups.

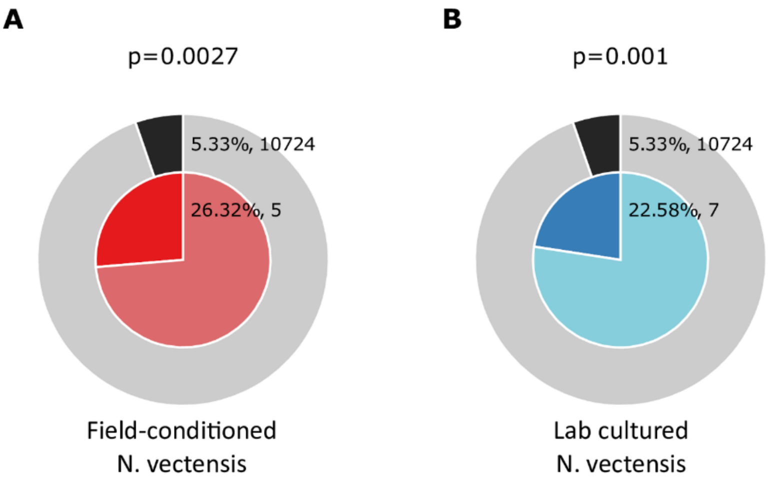

C

GO term ID	Description	Genes in term
GO:0090403	oxidative stress-induced premature senescence	ARNTL
GO:1903608	protein localization to cytoplasmic stress granule	YBX1
GO:0090400	stress-induced premature senescence	ARNTL
GO:0033555	multicellular organismal response to stress	NR2E1
GO:0090398	cellular senescence	ARNTL, SRF
GO:0043149	stress fiber assembly	SRF
GO:0030968	endoplasmic reticulum unfolded protein response	CREB3L4
GO:0043618	regulation of transcription from RNA polymerase II promoter in response to stress	E2F4
GO:0043620	regulation of DNA-templated transcription in response to stress	E2F4
GO:1990845	adaptive thermogenesis	FOXC2, ARNTL
GO:0034976	response to endoplasmic reticulum stress	CREB3L4
GO:0097193	intrinsic apoptotic signaling pathway	NKX3-1
GO:0034599	cellular response to oxidative stress	ARNTL
GO:0006979	response to oxidative stress	ARNTL
GO:0080135	regulation of cellular response to stress	NKX3-1
GO:0080134	regulation of response to stress	FOXC2, NKX3-1, ARNTL
GO:0033554	cellular response to stress	NKX3-1, CREB3L4, E2F4, ARNTL, TFDP1, SRF
GO:0006950	response to stress	NR2E1, FOXC2, NKX3-1, HHEX, CREB3L4, E2F4, MSX2, ARNTL, TFDP1, SRF, CREB1

Figure S2: Rearing conditions effect TF enrichment and transcriptional response during heat exposure. (A and B) Enrichment of motifs for stress-responsive transcription factors in promotors of differentially expressed genes. The outer pie chart shows the number and the percentage of genes in the background (the total number of genes in all terms) that are associated with the stress related terms (in black); the inner pie chart shows the number and the percentage of genes in the individual input gene list (genes corresponding to TF motifs that are enriched in promoters of differentially expressed genes) that are associated with the stress related terms. The *p*-value indicates whether the stress membership is statistically significantly enriched in the list. GO term IDs, descriptions, and TF gene names are detailed in (C). Gene names in red are enriched in field-conditioned animals, and those in blue are enriched in lab-cultured animals.

Table S4: pathway enrichment statistics—Annotations (Excel file).

Table S5: pathway enrichment statistics—enrichment (Excel file)

Table S6. ATAC-seq primer sequences used to label libraries.

Primer index	Primer barcode	Primer forward sequence
Global for primer	Ad1 noMX:	AATGATACGGCGACCACCGAGATCTACACTCGTCGGCAGCGTCAGATGTG
A	Ad2.1_TAAGGCGA	CAAGCAGAAGACGGCATACGAGATTCGCCTTAGTCTCGTGGGCTCGGAGATGT
B	Ad2.2_CGTACTAG	CAAGCAGAAGACGGCATACGAGATCTAGTACGGTCTCGTGGGCTCGGAGATGT
C	Ad2.3_AGGCAGAA	CAAGCAGAAGACGGCATACGAGATTTCTGCCTGTCTCGTGGGCTCGGAGATGT
D	Ad2.4_TCCTGAGC	CAAGCAGAAGACGGCATACGAGATGCTCAGGAGTCTCGTGGGCTCGGAGATGT
E	Ad2.5_GGACTCCT	CAAGCAGAAGACGGCATACGAGATAGGAGTCCGTCTCGTGGGCTCGGAGATGT
F	Ad2.6_TAGGCATG	CAAGCAGAAGACGGCATACGAGATCATGCCTAGTCTCGTGGGCTCGGAGATGT
G	Ad2.7_CTCTCTAC	CAAGCAGAAGACGGCATACGAGATGTAGAGAGGTCTCGTGGGCTCGGAGATGT
H	Ad2.8_CAGAGAGG	CAAGCAGAAGACGGCATACGAGATCCTCTCTGGTCTCGTGGGCTCGGAGATGT
I	Ad2.9_GCTACGCT	CAAGCAGAAGACGGCATACGAGATAGCGTAGCGTCTCGTGGGCTCGGAGATGT
J	Ad2.10_CGAGGCTG	CAAGCAGAAGACGGCATACGAGATCAGCCTCGGTCTCGTGGGCTCGGAGATGT
K	Ad2.11_AAGAGGCA	CAAGCAGAAGACGGCATACGAGATTGCCTCTTGTCTCGTGGGCTCGGAGATGT
L	Ad2.12_GTAGAGGA	CAAGCAGAAGACGGCATACGAGATTCCTCTACGTCTCGTGGGCTCGGAGATGT
M	Ad2.13_GTCGTGAT	CAAGCAGAAGACGGCATACGAGATATCACGACGTCTCGTGGGCTCGGAGATGT
N	Ad2.14_ACCACTGT	CAAGCAGAAGACGGCATACGAGATACAGTGGTGTCTCGTGGGCTCGGAGATGT
O	Ad2.15_TGGATCTG	CAAGCAGAAGACGGCATACGAGATCAGATCCAGTCTCGTGGGCTCGGAGATGT
P	Ad2.16_CCGTTTGT	CAAGCAGAAGACGGCATACGAGATACAAACGGGTCTCGTGGGCTCGGAGATGT
Q	Ad2.17_TGCTGGGT	CAAGCAGAAGACGGCATACGAGATACCCAGCAGTCTCGTGGGCTCGGAGATGT
R	Ad2.18_GAGGGGTT	CAAGCAGAAGACGGCATACGAGATAACCCCTCGTCTCGTGGGCTCGGAGATGT
S	Ad2.19_AGGTTGGG	CAAGCAGAAGACGGCATACGAGATCCCAACCTGTCTCGTGGGCTCGGAGATGT
T	Ad2.20_GTGTGGTG	CAAGCAGAAGACGGCATACGAGATCACCACACGTCTCGTGGGCTCGGAGATGT
U	Ad2.21_TGGGTTTC	CAAGCAGAAGACGGCATACGAGATGAAACCCAGTCTCGTGGGCTCGGAGATGT
V	Ad2.22_TGGTCACA	CAAGCAGAAGACGGCATACGAGATTGTGACCAGTCTCGTGGGCTCGGAGATGT
W	Ad2.23_TTGACCCT	CAAGCAGAAGACGGCATACGAGATAGGGTCAAGTCTCGTGGGCTCGGAGATGT
X	Ad2.24_CCACTCCT	CAAGCAGAAGACGGCATACGAGATAGGAGTGGGTCTCGTGGGCTCGGAGATGT

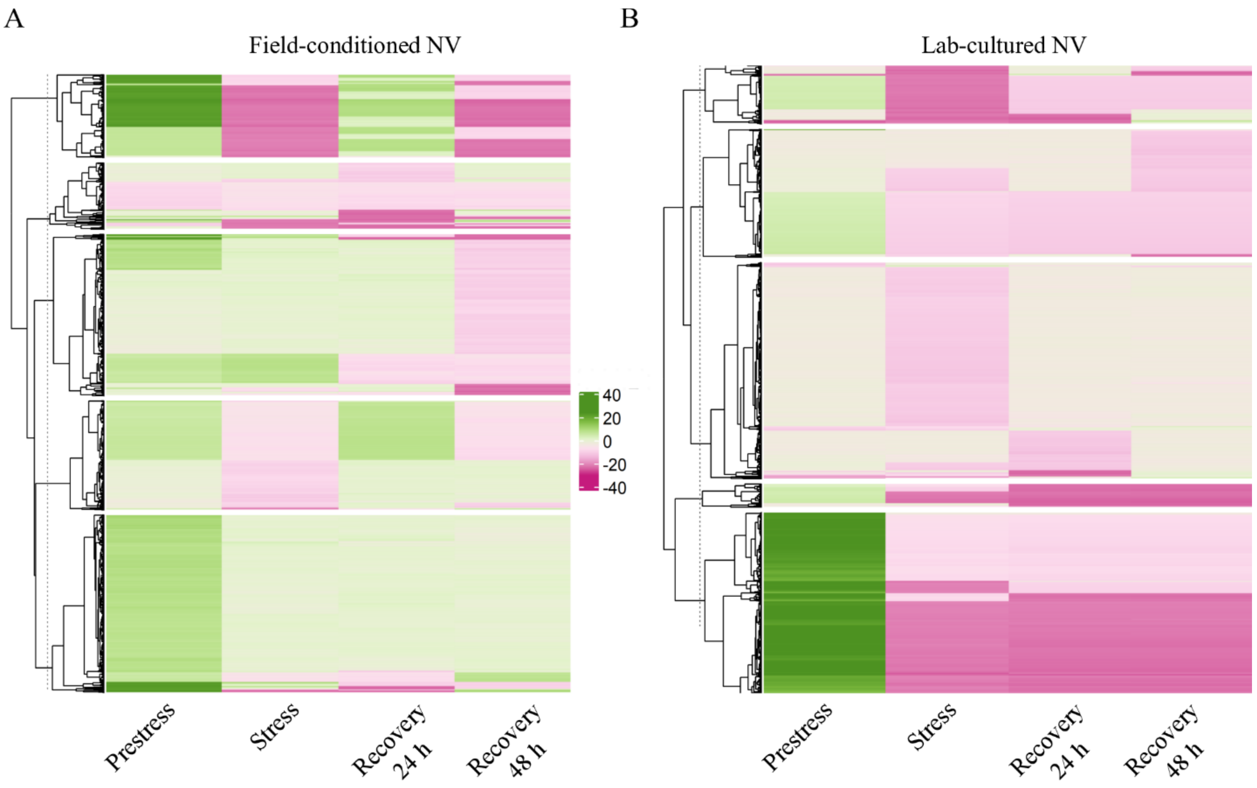

Figure S3: Dynamic chromatin changes during heat exposure in field-conditions and lab-cultured *N. vectensis* promoters. (A) 4462 differentially accessible promoters of field-conditioned *N. vectensis.* (B) 4633 differentially accessible promoters of lab-cultured *N. vectensis.*
